# Off-Label Use of Gabapentinoids in Non-diabetic Patients Referred to a Rheumatology Clinic: A Cross-Sectional Study

**DOI:** 10.7759/cureus.106080

**Published:** 2026-03-29

**Authors:** Gowri Renganathan, Sara Alhariri, Jesus Gutierrez, Nibesh Pathak, Shrilekha Sairam

**Affiliations:** 1 Department of Internal Medicine, Texas Tech University Health Sciences Center El Paso Paul L. Foster School of Medicine, El Paso, USA; 2 Department of Internal Medicine, University Medical Center of El Paso, El Paso, USA; 3 Department of Internal Medicine, University of Texas Health Science Center at San Antonio, San Antonio, USA; 4 Department of Internal Medicine, Institute of Medicine, Tribhuvan University, Kathmandu, NPL

**Keywords:** gabapentin, gabapentinoids, joint pain, low back pain, neuropathic pain, off-label prescription, opioids, pregabalin, psychotropics, tramadol

## Abstract

Background

Gabapentin is FDA-approved for postherpetic neuralgia and partial-onset seizures, while pregabalin is approved for several neuropathic pain conditions, fibromyalgia, and as adjunctive therapy for partial-onset seizures. Despite limited evidence for many off-label uses, gabapentinoids are frequently prescribed, raising concerns about adverse effects, misuse, and dependence. This study examines gabapentinoid prescribing patterns among patients referred to our rheumatology clinic.

Patients and methods

A cross-sectional study of patients without diabetes (N=466) was conducted among newly referred patients to our rheumatology clinic. Data was extracted from the electronic medical records system and recorded in the REDCap database. Patients' presenting complaints, comorbid conditions, and current medications were analyzed. Descriptive analyses were performed, and statistical analyses were conducted using a t-test for continuous variables, a chi-square test for categorical variables, and effect sizes were reported as odds ratios (OR).

Key results

Our findings reveal that 17.8% of patients referred to our clinic were prescribed gabapentinoids, many of whom also received concomitant prescriptions for opioids, including tramadol (34%), and antidepressants (32.5 %). Gabapentinoid use was more prevalent among patients with depression (OR=4.51) and anxiety (OR=3.69). Additionally, patients presenting with back pain (OR=3.78) and fatigue (OR=1.75) were more likely to be taking gabapentinoids. In contrast, symptoms such as multiple joint pain, dry eyes, and dry mouth showed no significant difference.

Conclusion

Our study provides insight into prescribing practices of gabapentinoids within the rheumatology setting. About one-third of the patients on gabapentinoids were also taking other medications with greater potential for harm with concomitant use. Evidence supporting the efficacy of gabapentinoids in different medical conditions is needed to guide clinicians.

## Introduction

The FDA has approved both gabapentin and pregabalin for the treatment of postherpetic neuralgia in adults and as adjunctive therapies for managing partial-onset seizures. Additionally, pregabalin is approved for neuropathic pain associated with diabetic peripheral neuropathy, fibromyalgia, and spinal cord injury-related neuropathic pain [1,2]. Among all prescriptions, off-label use of gabapentinoids is believed to constitute a significant portion. However, determining accurate prevalence rates at both national and global levels remains challenging [3].

Due to their diverse effects on the gamma-aminobutyric acid (GABA) system, gabapentinoids have been increasingly used for various conditions, including non-diabetic neuropathies, chronic back pain with or without radiculopathy, restless leg syndrome, chronic refractory migraines, preoperative pain, anxiety, attention deficit disorder, bipolar disorder, and alcohol withdrawal seizures, among others [3-6]. Despite being a relatively safer option, adverse events such as dizziness, fatigue, and memory difficulties highlight the importance of cautious prescribing [7]. While off-label drug use is legal and a common global practice, the increasing use of gabapentinoids raises concerns due to insufficient or low-quality evidence supporting their effectiveness in these cases [8,9]. Moreover, growing concerns exist regarding the potential misuse of gabapentinoids due to their risk of adverse effects, dependency, and abuse. This risk is further heightened when these drugs are co-prescribed with other central nervous system depressants, such as opioids [10]. 

We undertook a study to understand prescribing patterns and the prevalence of possible off-label use of gabapentinoids in patients newly referred to our rheumatology clinic. As it is approved for neuropathic pain, we assessed the prevalence in patients without diabetes. To better understand possible clinical symptoms and comorbid conditions associated with gabapentin use in practice, we focused on their prevalence in specific presenting symptoms. Concomitant use of gabapentinoid with opioids and psychotropic medications was evaluated to better understand the population at greater risk.

Objectives

Primary Objective

To determine the prevalence of gabapentinoid prescribing among new patients without diabetes referred to our rheumatology clinic.

Secondary Objective

To identify the presenting symptoms and comorbid conditions associated with gabapentinoid prescription in this population and to identify the frequency of co-prescription with pain medications, including nonsteroidal anti-inflammatory drugs, opiates, and antidepressants.

## Materials and methods

Study design

A cross-sectional study was conducted involving 466 adult non-diabetic patients who were newly referred to our rheumatology clinic between March 1, 2019, and February 1, 2020. The study received approval from the Institutional Review Board (IRB, #E20127). Reporting of this study followed the Strengthening the Reporting of Observational Studies in Epidemiology (STROBE) guidelines.

Study population

The study population included 466 patients, of whom 359 (77%) were women and 107 (23%) were men. The cohort consisted of 364 (78%) Hispanic patients, 79 (17%) White patients, and 19 (4%) African American patients.

Inclusion and exclusion criteria

The inclusion criteria were adult patients (≥18 years) who were non-diabetic and newly referred to the rheumatology clinic during the study period. Exclusion criteria included patients with a known diagnosis of diabetes mellitus, incomplete medical records, spinal cord injury, or postherpetic neuralgia.

Definition of off-label use

Off-label use of gabapentinoids was defined as prescriptions for indications other than those approved by the FDA. FDA-approved indications include postherpetic neuralgia and partial-onset seizures for gabapentin, and neuropathic pain associated with diabetic peripheral neuropathy, postherpetic neuralgia, fibromyalgia, and adjunctive therapy for partial-onset seizures for pregabalin [1-3]. Prescriptions documented for any other clinical indication were classified as off-label use.

Data collection

Data was extracted from the electronic medical record (EMR) system and entered into a REDCap database (Vanderbilt University, Nashville, TN, USA) for standardized analysis [11,12]. Demographic variables included age, sex, and race/ethnicity. The presenting complaints were obtained from referral documentation, from the history of presenting complaints, and from the assessment section of the first clinic visit notes. Comorbid conditions were identified using diseases listed in the past medical history and problem lists sections noted in the first clinic visit documentation. Medication data were obtained from the active medication list at the time of the initial rheumatology visit. Gabapentinoid use (gabapentin or pregabalin), as well as opioids and psychotropic medications, was specifically recorded.

Statistical analysis

Descriptive statistics were used to summarize patient demographics, clinical characteristics, and medication use patterns. Continuous variables were analyzed using Student’s t-test, and categorical variables were compared using the chi-square test. Odds ratios (ORs) with corresponding 95% confidence intervals were calculated to estimate effect sizes. A p-value <0.05 was considered statistically significant. A univariate analysis was performed without adjustment for confounding factors.

## Results

Among the new patients referred, 466 were non-diabetics and 83 (17.8%) were on gabapentinoids. The prevalence of gabapentin or pregabalin use in this population was 83 (17.8%). We observed no significant differences in gender, race, or weight among those prescribed gabapentinoids compared to those who were not.

**Figure 1:**
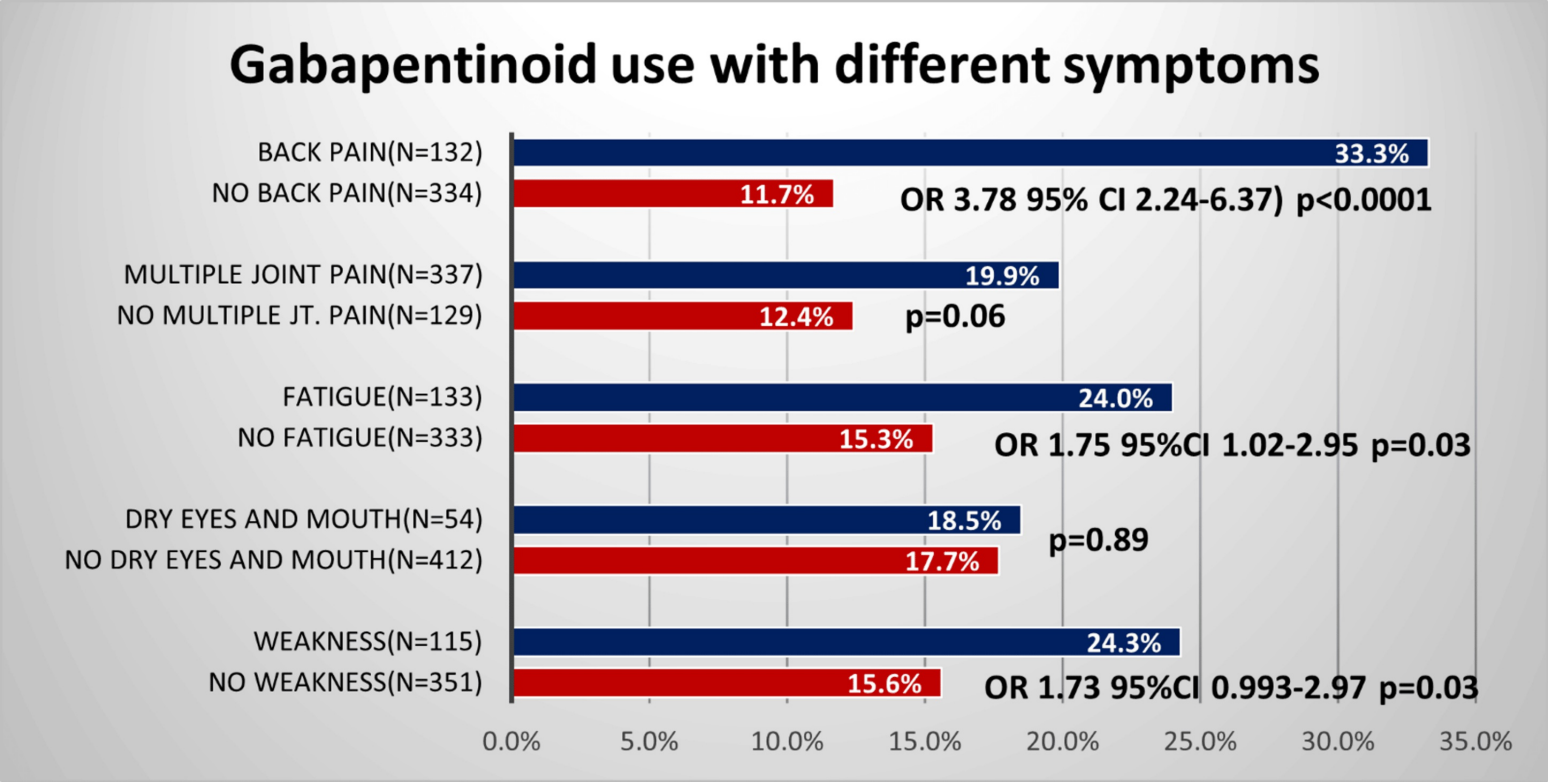
The prevalence of gabapentin use in patients presenting with specific clinical symptoms. Higher prevalence was noted in patients presenting with back pain (p<0.0001) and with fatigue (p=0.03)

The most common presenting symptom in patients referred was multiple joint pain (72%). The use of gabapentinoids among patients varied significantly by certain clinical presentations and comorbidities, as shown in Table 1 and Figure 1. Notably, gabapentinoid use was significantly associated with back pain (odds ratio (OR) 3.78, 95% confidence interval (CI) 2.24-6.37, p<0.0001) and fatigue (OR 1.75, 95%CI 1.02-2.95, p=0.03). Gabapentinoid use did not differ in patients who presented with or without multiple joint pain or dryness of the eyes and mouth. Although gabapentinoid prescription was more prevalent among those with weakness (p=0.03), the 95% confidence interval included the value 1.

**Table 1:** Presenting complaints, comorbid states, and medication use in patients on gabapentinoids and those not taking gabapentinoids. NSAIDs: Non-steroidal anti-inflammatory drugs.

Variable	Gabapentinoid Users (N=83)	Not Taking Gabapentinoids (N=383)	Odds Ratio (OR)	95% Confidence Interval (CI)	p-value	Chi-square (χ²)
Presenting Complaints						
Back pain (N=132)	44	88	3.78	2.24-6.30	<0.0001	16.0
Multiple joint pain (N=337)	67	270	1.75	0.95-3.38	0.06	3.5
Fatigue (N= 133)	32	101	1.75	1.02-2.95	0.03	4.7
Dry eyes and mouth (N=54)	10	44	1.10	0.45-2.25	0.89	0.02
Weakness (N=115)	28	87	1.73	0.99-2.97	0.03	4.7
Comorbid Conditions						
Depression (N=53)	23	30	4.51	2.32-8.62	<0.0001	19.0
Anxiety (N=42)	17	25	3.69	1.76-7.54	<0.0001	15.7
Hypertension (N=141)	31	110	1.49	0.87-2.49	0.12	2.4
Thyroid Disease (N=64)	14	50	1.35	0.65-2.65	0.36	0.8
Medication Use						
Opiates (other than tramadol) (N=23)	8	15	2.62	0.92-6.84	0.0291	4.8
Tramadol (N=42)	20	22	5.21	2.52-10.61	<0.0001	18.5
NSAIDs (N=163)	32	141	1.64	0.97-2.72	0.0426	4.1
Antidepressants (N=74)	27	47	3.45	1.89-6.17	<0.0001	17.0

**Figure 2:**
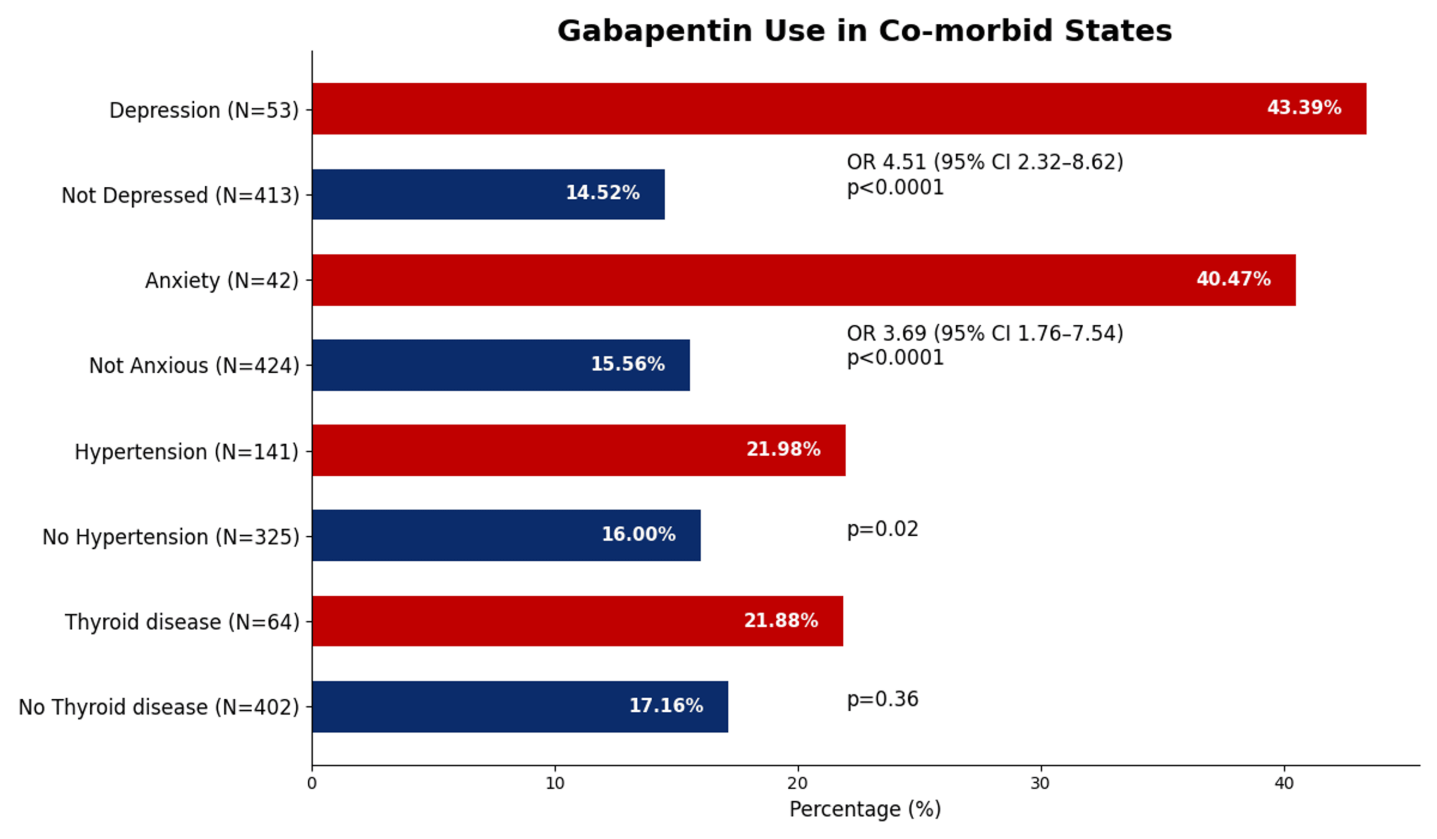
The prevalence of gabapentinoid use in patients with different comorbid states. Significantly higher prevalence of gabapentinoid use was noted in those with depression and anxiety (p<0.0001)

A diagnosis of depression was noted in 53 patients (11.37% of the study population) and was significantly associated with gabapentinoid prescription. About 43.39% of patients with depression were on gabapentinoids compared to 14% of non-depressed patients (OR 4.51, 95%CI: 2.32-8.62, p<0.0001) as mentioned in Table 1 and Figure 2. Anxiety diagnosis was recorded in 9.01% (n=42) of the cohort. Gabapentinoid use was markedly higher in these patients compared to those without anxiety (40.47% vs. 15.5%; OR 3.68, 95% CI: 1.76-7.54, p<0.0001). The prevalence of gabapentinoid use in different comorbidities is shown in Table 1 and Figure 2.

**Figure 3:**
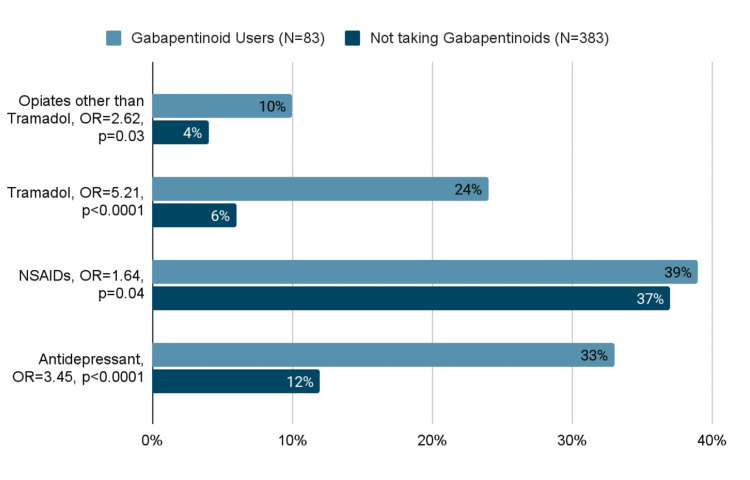
The frequency of other medication use among patients for gabapentinoid prescriptions among patients taking other medications. P<0.05 for all the mentioned prescriptions.

Concomitant medication use among gabapentinoid users (N=83) was assessed, and 10% were taking opiates other than tramadol, 24% were taking tramadol, and 32.5% were on antidepressants. Patients on tramadol were more likely to be prescribed gabapentinoids than those not on tramadol (47.6% vs. 15.5%; OR, 5.21; p<0.0001). Similarly, antidepressant use was strongly associated with gabapentinoid prescription (OR 3.45, p<0.0001). No difference was observed with non-steroidal anti-inflammatory drug (NSAID) use. Table 1 and Figure 3 list the concomitant medications used in this study population.

## Discussion

Several studies have shown limited evidence supporting the efficacy of gabapentinoids for many of the disorders for which they are prescribed off-label. However, gabapentinoid use remains frequent. As gabapentinoids are approved for neuropathic pain and diabetic pain neuropathy, and as we were focusing on their off-label use, we chose those without diabetes as our study population.

We found 17.8 % of the patients without diabetes referred to us were prescribed gabapentinoids. The majority of the patients were referred for multiple joint pains.

In our study, gabapentin use was more common among patients with back pain (OR 3.78). Gabapentinoids have demonstrated efficacy in neuropathic pain [13], with some case reports and open-label studies suggesting potential benefits for low back pain. However, randomized, placebo-controlled trials supporting their efficacy in low back pain are lacking [14,15]. Among patients with chronic low back pain, 16%-35% have a neuropathic etiology [16]. While one trial comparing gabapentin with placebo reported pain relief in sciatica, a systematic review and meta-analysis by Giminez-Campos et al. did not provide clear evidence of the effectiveness of pregabalin and gabapentin for sciatica pain management [17]. In England, gabapentinoid prescriptions increased by 46% between 2011 and 2013 for back pain [7]. In our study, 33% of patients with back pain were taking gabapentinoids.

A study by Shanthanna et al. found that gabapentinoid use in chronic low back pain was associated with a higher incidence of adverse events compared to placebo, including dizziness, fatigue, cognitive difficulties, and visual disturbances [7]. Our study found an association between gabapentinoid use and symptoms of fatigue and numerically higher use in patients presenting with weakness. However, it remains unclear whether patients experiencing these symptoms were more likely to be prescribed gabapentinoids or whether those taking gabapentinoids reported these symptoms more frequently.

About 41%-43% of patients with comorbid states, depression, or anxiety in our study, the majority of whom were referred for multiple joint pain, were prescribed gabapentinoids. A U.S.-based survey using the TriNetX electronic health records network found that gabapentin had been prescribed at least once in 13.6% of patients with bipolar disorder, 12.7% with insomnia disorder, and 11.5% with anxiety disorder. For pregabalin, the corresponding figures were 2.9% for bipolar disorder, 3% for insomnia disorder, and 2.6% for anxiety disorder [18]. A study found that among off-label psychiatric indications, depressive disorders were the most frequent diagnosis (5.3%) [19]. A systematic review of the use of gabapentin in psychiatric and substance use disorders found no evidence supporting its efficacy in bipolar disorder, though there was some indication of efficacy for certain anxiety disorders [19,20]. While efficacy for generalized anxiety disorder was noted [21], its effectiveness in other anxiety disorders, such as social anxiety disorder, remains unclear [22]. In our study, patients with anxiety or depression were more likely to be prescribed gabapentinoids (OR 3.68 and 4.51, respectively).

In the general population, gabapentinoid abuse has a prevalence of 1.6%, with rates ranging from 3% to 68% among opioid abusers. An international adverse event database identified 11,940 reports of gabapentinoid abuse between 2004 and 2015, with over 75% of cases reported after 2012. The risk factors for misuse include a history of substance abuse, particularly opioid use, and psychiatric comorbidities [23,24]. In our study, patients who were on opiates and tramadol were more likely to be prescribed gabapentinoids (OR 2.62 and 5.21, respectively).

A large Canadian study found that approximately 8% of patients prescribed opioids were also concurrently prescribed gabapentin, a combination linked to a 50% increase in opioid-related mortality risk [10]. Similarly, studies in the United States and UK indicate that 15%-22% of individuals with opioid use disorder also misuse gabapentin [25]. Among patients referred to our center, 47% of those on tramadol were also prescribed gabapentin. Gabapentinoid use has been associated with drug-induced respiratory depression, either when used alone or in combination with other medications [26].

Strengths

The use of real-world electronic medical record data from consecutive new referrals provides insight into current practice in gabapentinoid prescription among patients, most of whom were referred for pain. A reasonably large sample (N=466) and a single-center study favored the detection of clinically meaningful associations. Reporting of effect sizes (odds ratios) alongside p-values aids clinical interpretation.

Limitations

This retrospective, single-center, cross-sectional study has several important limitations. To identify off-label use, we excluded patients with diabetes who may have neuropathy. However, not all patients with diabetes would have neuropathic pain, resulting in selection bias. Data were extracted subjectively from recorded information in clinic notes, and thus, there was potential for inaccuracy due to inadequate recording. The observed associations may have excluded unmeasured factors and clinical details not captured in the record. Its design and statistical analysis limit control of confounding factors, leading to weaker causal inference. Inclusion of only patients referred to and evaluated at our clinic introduces referral/selection bias and greatly limits generalizability. Medication indications and histories were abstracted from clinical notes rather than being prospectively collected or derived from pharmacy fill records, introducing potential recall/misclassification bias and preventing reliable measurement of adherence to gabapentinoid therapy. We lacked longitudinal outcome data to assess effectiveness, harms, or dependence, and our analyses were unadjusted for many potential confounders, raising the possibility that the reported odds ratios over- or underestimate the true associations.

## Conclusions

In our single-center cross-sectional study, 17.8% of new rheumatology patients without diabetes were prescribed gabapentinoids, with a higher likelihood among those presenting with back pain or fatigue. Patients with depression or anxiety were more frequently taking gabapentinoids. Concurrent opioid (including tramadol) and antidepressant prescriptions were 34% and 32.5%. These findings raise concern about frequent off-label use in this setting and the potential for harm from high-risk co-prescribing. With limited evidence for efficacy in many clinical indications, we need to reassess the role of gabapentinoids and current practice in managing chronic pain and psychiatric comorbidity.
